# Clinical efficacy and safety of Tripterygium wilfordii Hook in the treatment of diabetic kidney disease stage IV: A meta-analysis of randomized controlled trials: Erratum

**DOI:** 10.1097/MD.0000000000015486

**Published:** 2019-04-26

In the article, “Clinical efficacy and safety of Tripterygium wilfordii Hook in the treatment of diabetic kidney disease stage IV: A meta-analysis of randomized controlled trials”,^[1]^ which appears in Volume 98, Issue 11 of *Medicine*, affiliation b should be “Grade 2016, the Second Clinical Medical College of Nanchang University, China.”
